# *In vivo* modification of tyrosine residues in recombinant mussel adhesive protein by tyrosinase co-expression in *Escherichia coli*

**DOI:** 10.1186/1475-2859-11-139

**Published:** 2012-10-24

**Authors:** Yoo Seong Choi, Yun Jung Yang, Byeongseon Yang, Hyung Joon Cha

**Affiliations:** 1 Department of Chemical Engineering, Chungnam National University, Daejon, 305-764, Korea; 2 Department of Chemical Engineering, Pohang University of Science and Technology, Pohang, 790-784, Korea; 3 Ocean Science and Technology Institute, Pohang University of Science and Technology, Pohang, 790-784, Korea

**Keywords:** Mussel adhesive protein, Dopa, Dopaquinone, *In vivo* modification, Tyrosinase, Co-expression, *Escherichia coli*

## Abstract

**Background:**

In nature, mussel adhesive proteins (MAPs) show remarkable adhesive properties, biocompatibility, and biodegradability. Thus, they have been considered promising adhesive biomaterials for various biomedical and industrial applications. However, limited production of natural MAPs has hampered their practical applications. Recombinant production in bacterial cells could be one alternative to obtain useable amounts of MAPs, although additional post-translational modification of tyrosine residues into 3,4-dihydroxyphenyl-alanine (Dopa) and Dopaquinone is required. The superior properties of MAPs are mainly attributed to the introduction of quinone-derived intermolecular cross-links. To solve this problem, we utilized a co-expression strategy of recombinant MAP and tyrosinase in *Escherichia coli* to successfully modify tyrosine residues *in vivo*.

**Results:**

A recombinant hybrid MAP, fp-151, was used as a target for *in vivo* modification, and a dual vector system of pET and pACYC-Duet provided co-expression of fp-151 and tyrosinase. As a result, fp-151 was over-expressed and mainly obtained from the soluble fraction in the co-expression system. Without tyrosinase co-expression, fp-151 was over-expressed in an insoluble form in inclusion bodies. The modification of tyrosine residues in the soluble-expressed fp-151 was clearly observed from nitroblue tetrazolium staining and liquid-chromatography-mass/mass spectrometry analyses. The purified, *in vivo* modified, fp-151 from the co-expression system showed approximately 4-fold higher bulk-scale adhesive strength compared to *in vitro* tyrosinase-treated fp-151.

**Conclusion:**

Here, we reported a co-expression system to obtain *in vivo* modified MAP; additional *in vitro* tyrosinase modification was not needed to obtain adhesive properties and the *in vivo* modified MAP showed superior adhesive strength compared to *in vitro* modified protein. It is expected that this co-expression strategy will accelerate the use of functional MAPs in practical applications and can be successfully applied to prepare other Dopa/Dopaquinone-based biomaterials.

## Background

To live in tidal aqueous environments and to protect themselves from predators, marine sessile organisms, such as mussels and tubeworms, attach themselves to hard substratum using protein-based adhesives [1,2]. Marine-derived protein bioadhesives are considered promising biomaterials in medical, environmental, and industrial applications because of their versatile adhesive properties, such as strong and flexible adhesion, durability, biodegradability, and biocompatibility [2,3]. Remarkably, these properties are mainly attributable to the introduction of quinone-derived intermolecular cross-links among individual adhesive proteins (quinone tanning) [4]. Hydroxylation of tyrosine residues leads to the formation of 3,4-dihydroxyphenyl-alanine (Dopa), which plays an important role in the adhesion and cross-linking of a catecholic precursor. Dopa enables adhesive proteins to cross-link *via* aryl coupling, thiol addition, coordination of multiple ligands by Fe^3+^, and chemisorption by chelation of surface metal oxides [5]. High levels of Dopa content are observed in mussel adhesive proteins (MAPs; 3~30 mol%) [6] and tubeworm cement proteins (7~10 mol%) [7].

MAPs have been extensively studied over the last 30 years as a characteristic example of Dopa-based biological adhesion. Six distinct adhesive proteins of type 1 (fp-1) through type 6 (fp-6) have been identified from adhesive plaque, and the proteins can be extracted by acid-based chemical extraction processes [4,6]. However, the extracted amount is not practically applicable for bioadhesives as 10,000 mussels are required to obtain ~1 g of the most extractable MAP fp-1, and at least 100 mg of protein is needed for small, conventional adhesive tests, such as tensile or shear strength analysis, for obtaining mechanical properties of adhesive biomaterials [2,8]. Thus, recombinant MAP expression systems have been applied to overcome the low production yield of natural extraction, but post-translational modifications, such as Dopa and Dopaquinone conversion, do not always occur naturally in recombinant systems [2,6]. Especially, recombinant MAPs from high yield bacterial expression systems require additional modifications to become functional. The modification of tyrosine residues can be accomplished *in vitro* with mushroom tyrosinase [9]. Some studies have shown that the levels of Dopa are related to the adhesive properties [10-12]. MAPs tend to easily aggregate in solution due to their high levels of aromatic and basic amino acids [13-15]. In addition, tyrosinase is known to most effectively oxidize free tyrosines rather than those bound within large protein and tyrosine residues within monomeric protein rather than protein aggregate [16,17]. Thus, the *in vitro* modification yield is relatively low due to limited access of the tyrosine residues within MAP [12]. To improve the modification efficiency, the modification with tyrosinase needs to be conducted as soon as the protein is expressed *in vivo*. Thus, an *in vivo* modification system for tyrosine residues, such as natural MAP production in mussel, is required to prepare recombinant MAPs in bacterial expression systems.

In the present work, for the first time, we co-expressed functional tyrosinase with recombinant MAP in *Escherichia coli* to efficiently modify tyrosine residues *in vivo*. The recombinant hybrid MAP fp-151 [18], which is composed of six fp-1 decapeptide repeats at both termini of fp-5, was used as a model. Previously, high production yield (~1 g-purified protein per 1 L-pilot-scale fed-batch culture) and improved solubility (>300 g/L) of fp-151 showed strong potential as a practical bioadhesive [18].

## Results and discussion

### Co-expression of fp-151 and tyrosinase

**Figure 1:**
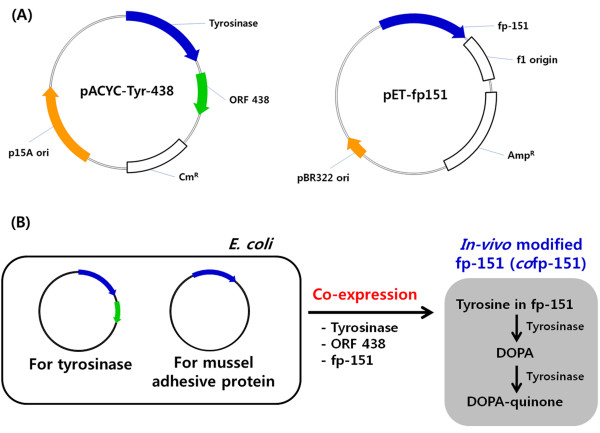
**Schematic diagram of the co-expression system to obtain *in vivo *tyrosine-modified MAP.** (**A**) pACYC-Tyr-438 harboring two genes of tyrosinase and ORF 438 was used for functional expression of tyrosinase (left vector map), and pET-fp151 was used for expression of fp-151 (right vector map). (**B**) The two recombinant plasmids were introduced into *E. coli* BL21 (DE3) for co-expression of fp-151, tyrosinase, and ORF 438. Through this co-expression strategy, *in vivo* tyrosine-modified MAP can be obtained without additional *in vitro* modification.

Previously, we successfully over-expressed fp-151 (~40% of total protein) using a pET vector system with strong T*7* promoter and pBR322 origin (right vector map, Figure 1A) [18]. Because the pET vector can be used in combination with a vector system with p15A replicon [19] and functional expression of tyrosinase from *Streptomyces antibioticus* was reported in *E. coli* under the control of T*7* promoter [20], recombinant pACYC-Tyr-438 plasmid (left vector map, Figure 1A) was prepared from pACYCDuet-1, which carries the p15A origin. For functional expression of recombinant tyrosinase, ORF 438, which is essential for copper incorporation into the active site of tyrosinase [21], was also co-expressed under the T*7* promoter. Then, both recombinant pET-fp151 and pACYC-Tyr-438 plasmids were introduced into *E. coli* BL21(DE3) to obtain *in vivo* tyrosine-modified fp-151 (Figure 1B).

**Figure 2:**
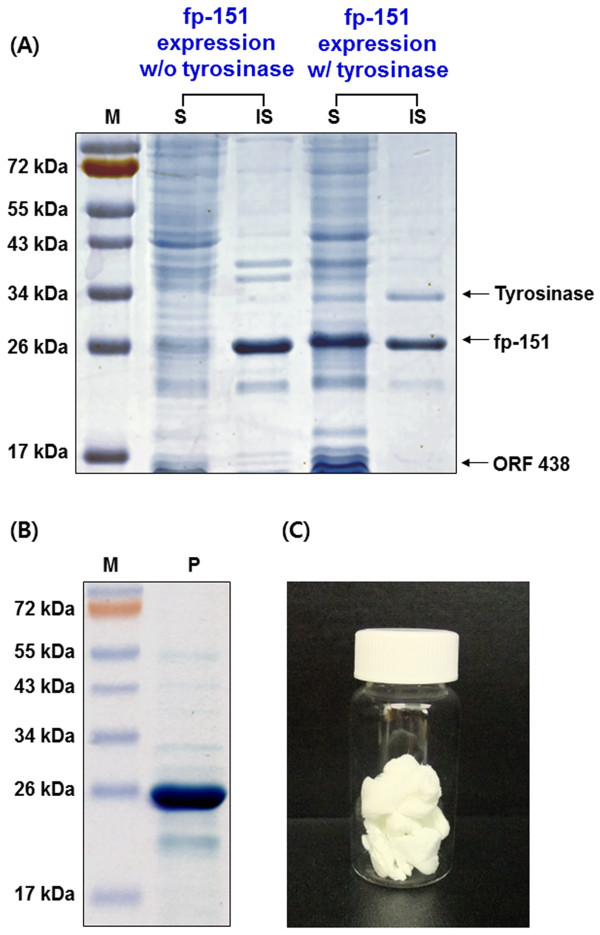
**Over-expression and purification of *co*fp-151.** (**A**) SDS-PAGE analysis for the expression of fp-151 without and with tyrosinase. The fp-151 protein was expressed alone using the pET-fp151 plasmid (fp-151 expression w/o tyrosinase) or co-expressed using the two plasmids pACYC-Tyr-438 and pET-fp151 (fp-151 expression w/ tyrosinase) in *E. coli*. Lanes: M, protein molecular weight marker; S, soluble supernatant fraction; IS, insoluble cell debris fraction. (**B**) SDS-PAGE analysis of *co*fp-151 purified by Ni-NTA affinity chromatography. Lanes: M, protein molecular weight marker; P, purified sample. (**C**) Lyophilized powder of the purified *co*fp-151.

Recombinant fp-151 was successfully co-expressed with tyrosinase and ORF 438 in the dual vector system (Figure 2A). We found that its expression level was similar to that of fp-151 from pET-fp151 alone. In addition, the growth of *E. coli* in the co-expression system displayed a similar trend to that of *E. coli* in the sole fp-151expression system (data not shown). Thus, the co-expression system does not seem to harm the growth of the host. Interestingly, the co-expressed fp-151 (*co*fp-151) was observed in both the soluble (mainly) and insoluble fractions, whereas the fp-151 alone was mostly expressed in the form of insoluble inclusion bodies. The soluble fp-151 in SDS-PAGE was separated at a higher molecular weight than its calculated molecular mass (~24 kDa), which was also observed in previous reports and other recombinant MAPs [18,22,23]. Tyrosinase (~32 kDa) in the dual vector system was also co-expressed both as soluble and insoluble forms (Figure 2A), although the expression level was significantly decreased compared to that from *E. coli* BL21(DE3) bearing the pACYC-Tyr-438 plasmid only; tyrosinase from the pACYC-Tyr-438 alone was obtained in insoluble inclusion bodies at 37°C culture (~45% of total protein) and as a soluble protein at 20°C culture (~15% of total protein) after IPTG induction (data not shown). ORF 438 (~15 kDa) was only expressed in a soluble form in the dual vector system (Figure 2A). We surmised that soluble expression of recombinant fp-151 in the dual vector system might be from its modification by functional tyrosinase co-expression.

### Purification of *co*fp-151

Affinity chromatography was conducted to purify the *co*fp-151 from the soluble fraction. Affinity purification was not successful under native conditions because the *co*fp-151 bound poorly to the Ni-NTA resin (data not shown). When gel filtration chromatography was applied, a group of the largest molecules was separated as a high resolution fraction from the remainder of the sample. However, this fraction was a complex mixture whose main component was *co*fp-151 (data not shown). The *co*fp-151 protein might be bound to other components in soluble *E. coli* lysate by its adhesive properties, such as modified tyrosine residues, hydrogen bond-forming residues, and the unique conformation of adhesive proteins. Thus, affinity purification was conducted again under denaturing conditions using 8 M urea, and we found that the *co*fp-151 was able to bind to the resin. Unfortunately, the *co*fp-151 was eluted poorly under normal elution conditions for hexahistidine (His_6_)-tagged proteins, such as 100–250 mM imidazole or weak acidic conditions of pH 4.5-5.3 (data not shown). Under these conditions, most of the target proteins remained bound to the column resin, but an increased concentration of imidazole and decreased pH conditions improved the elution yield of the purified protein. Because the purified *co*fp-151 protein precipitated during the dialysis step to remove the imidazole, the elution was optimized using acidic conditions. Finally, we obtained *co*fp-151 with above 95% purity by elution with 0.5 M HCl (Figure 2B). After dialysis using distilled water, the purified sample was lyophilized and stored at −80°C (Figure 2C).

### Analyses of tyrosine modification in *co*fp-151

**Figure 3:**
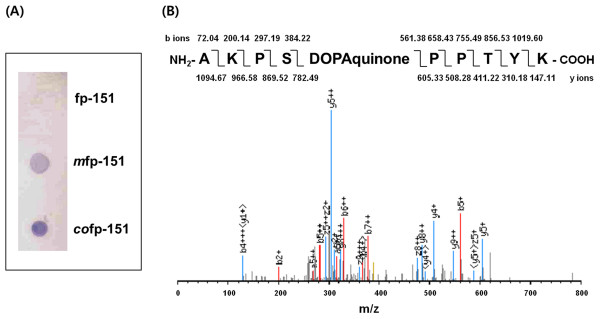
**Analyses of tyrosine modifications in *co*fp-151.** (**A**) NBT staining of unmodified fp-151 (fp-151), *in vitro* modified fp-151 using tyrosinase (*m*fp-151), and co-expressed fp-151 (*co*fp-151). (**B**) LC-MS/MS analysis of *co*fp-151.

Quinoproteins, which contain quinones and related quinonoid substances such as Dopaquinone and Dopa, are specifically stained by nitroblue tetrazolium (NBT) and glycinate solution because of their redox-cycling, although the intensity is different from quinones and quinonoid compounds [24]. Thus, we first investigated the *in vivo* modification of tyrosine residues in *co*fp-151 using the NBT/glycinate staining method. Along with non-modified fp-151 as a negative control and *in vitro* tyrosine-modified fp-151 (*m*fp-151) as a positive control, *in vivo* tyrosine-modified *co*fp-151 was dropped onto a nitrocellulose membrane and stained using NBT/glycinate solution. We found that non-modified fp-151 was not stained at all, but blue-purple spots were clearly shown in the *m*fp-151 and *co*fp-151 samples (Figure 3A), indicating the tyrosine residues of *co*fp-151 were successfully *in vivo* modified into quinones or quinonoid compounds by co-expression of tyrosinase. Importantly, the *co*fp-151 spot had a higher intensity than the *m*fp-151 spot, indicating the *in vivo* modification efficiency is higher than the *in vitro* modification efficiency. Next, we performed liquid-chromatography-mass/mass spectrometry (LC-MS/MS) analysis using a trypsin digest to confirm the presence of quinone relatives in the *co*fp-151 protein. From the LC-MS/MS analysis, Ala-Lys-Pro-Ser-Dopaquinone-Pro-Pro-Thr-Tyr-Lys fragments were detected, which can be from the repeating decapeptide unit of the fp-1 region in the hybrid MAP fp-151 (Figure 3B). However, Dopa residues were not found in the analysis. These results demonstrated that tyrosines at position 5 in the decapeptide unit of the fp-1 region in the *co*fp-151 were *in vivo* modified into Dopaquinone through co-expression of tyrosinase, although the modification of tyrosine residues was more frequently detected at position 9 than position 5 in the natural fp-1 [25]. Previously, the tyrosine modification at position 5 was observed in synthetic fp-1 decapeptide conversion using mushroom tyrosinase [9] and was also observed with *in vivo* modification of fp-151 in insect Sf9 cells [26]. Thus, the altered preference of hydroxylation position might be due to the different modification enzymes and/or the different structural features of fp-151.

In mussel, it is thought that Dopa residues are formed from post-translational modifications catalyzed by polyphenol oxidases [2,27]. Polyphenol oxidases catalyze the *o*-hydroxylation of monophenol to *o*-diphenols (catechols) and also further catalyze the dehydrogenation of *o*-diphenols to *o*-quinones. However, only Dopa has been observed in natural MAPs, and very little information about these enzymes exists. Thus, the conversion mechanism of Dopa molecules from tyrosine residues has yet to be clearly explained. Antioxidant molecules might block Dopa oxidation into Dopaquinone, or undiscovered polyphenol oxidase with high substrate specificity for tyrosine residues (or tyrosine hydroxylase) might be involved in the preparation of Dopa-incorporated MAPs. To the best of our knowledge, polyphenol oxidases have not been identified yet in mussel. Thus far, catechol oxidase activity was measured in the Golgi complex of *Mytilus edulis* and whole byssus structures [28,29], and nonhomogeneous catechol oxidases were only isolated from the mussel foot and regions of the byssus [30,31].

### Bulk adhesive strength of *co*fp-151

**Figure 4:**
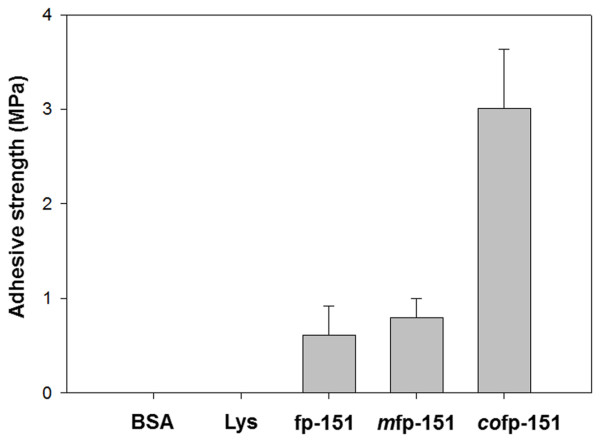
**Bulk-scale adhesive strength of *co*fp-151.** BSA, lysozyme (Lys), non-modified fp-151 (fp-151) and *m*fp-151 were utilized in the control experiments. The aluminum adherends were incubated at 37°C for 3 h in air before the measurements. Each adhesion measurement was repeated at least 5 times and averaged for a given sample.

We expected that the successful *in vivo* modification of tyrosine residues would demonstrate the superior adhesive properties of MAPs *via* quinone-derived intermolecular cross-linking. To this end, the bulk-scale adhesive strength of *co*fp-151 was measured using aluminum adherends due to their ease of use and good surface reproducibility. The adhesive strength of *m*fp-151 was also measured to compare the strengths of *in vitro* and *in vivo* tyrosine-modified fp-151. Bovine serum albumin (BSA) was used as a negative control. Non-modified fp-151 and lysozyme, which is a basic protein similar to fp-151 [32], were also used as controls. The aluminum adherends were attached under the same conditions using BSA, lysozyme, fp-151, *m*fp-151, and *co*fp-151, and the samples were incubated at 37°C for 3 h in air. Under these conditions, the shear strength of *co*fp-151 (3.01 ± 0.62 MPa) was 4-fold higher than that of *m*fp-151 (0.80 ± 0.20 MPa) (Figure 4). The observed shear strength difference of *in vivo* and *in vitro* tyrosine-modified fp-151 samples may come from the usage of different kinds of tyrosinase. However, MAPs tend to aggregate in neutral pH conditions [13-15] and treating higher concentrations of tyrosinase did not improve *in vitro* modification efficiency (data not shown). It was surmised that the steric hindrance by MAP aggregation could restrict the enzymatic reaction regardless of the types of tyrosinase. Thus, the strategy of *in vivo* modification through tyrosinase co-expression can overcome the relatively-low-yield problem observed in the *in vitro* tyrosinase-treated modification. It is important to note that *co*fp-151 showed significantly strong adhesive property although the tyrosinase co-expression mainly produced Dopaquinone. It is now accepted that Dopa residues are mainly involved in the remarkable adhesion and the Dopaquinone, which is converted from Dopa, is responsible for the cross-linking [5,11,33]. Thus, the quinone-based cross-linking/coupling *via* radical generation, imine formation, and Michael adduct formation contributes to the strong adhesion [33]. Until now, limited data of the bulk-scale adhesive strengths of MAPs have been reported; overall, adhesive strengths of 0.3~2 MPa were measured for mussel foot extract, synthetic repeats of fp-1 decapeptides, and *in vitro* tyrosinase-treated recombinant MAPs [12,18,34-38]. The formulation of MAPs through complex coacervation only showed adhesive strength above 3 MPa [35]. Thus, the preparation of MAPs using the co-expression strategy for *in vivo* modification was successful in obtaining MAPs with strong adhesive properties.

## Conclusions

Here, we produced a quinone-incorporated recombinant MAP, fp-151, in *E. coli*. Quinone residues were successfully incorporated into fp-151 by the co-expression of fp-151 and tyrosinase, which enhanced the adhesive strength of fp-151 significantly over that of *in vitro* modified fp-151. This approach can overcome the limitations of conventional *in vitro* tyrosinase modification of MAPs by directly preparing tyrosine-modified MAPs from *E. coli* without any additional modification steps after protein purification. Ultimately, we expect that MAPs will be used as bioadhesives with strong adhesive properties and good biocompatibility. The strategy used in this study will be expanded to prepare other Dopa/Dopaquinone-based adhesive materials including MAPs and accelerate the use of MAPs in practical applications, especially in various biomedical and tissue engineering fields.

## Methods

### Strains and vector construction

*E. coli* TOP10 (Invitrogen, Carlsbad, CA, USA) was used as a host for recombinant vector preparation and *E. coli* BL21 (DE3) cells (Merck KGaA, Darmstadt, Germany) were used for the co-expression of recombinant fp-151 and both tyrosinase and ORF 438 from *S. antibioticus*. *E. coli* cells were grown in Luria-Bertani (LB) medium with 50 μg/mL ampicillin (Sigma, St. Louis, MO, USA). *S. antibioticus* was obtained from the Korean Collection for Type Culture (DaeJeon, Korea). A single colony of *S. antibioticus* was grown in MYG broth (1% (w/v) malt extract, 0.4% (w/v) yeast extract, and 0.4% (w/v) glucose; pH 7.2) at 30°C. A genomic DNA of *S. antibioticus* was prepared using genomic DNA purification kit (Promega, Madison, WI, USA). The tyrosinase gene was amplified by polymerase chain reaction (PCR) using two primers of pSA-tyr-5p: 5’-cac ca*G GAT CC*g acc gtc cgc aag aac-3’ and pSA-tyr-3p: 5’-cac *AAG CTT* tca gac gtc gaa ggt-3’. The amplified product was digested with *Bam*HI and *Hind*III. Similarly, the ORF 438 gene was amplified by PCR using two primers of pSA-438-5p: 5’- cac *CAT ATG* ccg gaa ctc acc cgt-3’ and pSA-438-3p: 5’- cac *CTC GAG* tca gtt gga ggg gaa-3’, and the amplified product was digested using *Nde*I and *Xho*I. The digested products were introduced into pACYCDuet-1 plasmid (Merck KGaA, Darmstadt, Germany) using the same restriction enzyme sites. Finally, the introduced DNA sequences were confirmed by sequencing, and the functional tyrosinase expression vector was named pACYC-Tyr-438. In addition, the fp-151 gene was amplified from pENG151 [18] using two primers from the T*7* promoter primer: 5’-taa tac gac tca cta tag g-3’ and p151-NS-His-3pL 5’-cgc *AAG CTT* gta cgt tgg ag-3’. The amplified product was introduced into the *Nde*I and *Hind*III restriction sites of pET-23b (Merck KGaA, Darmstadt, Germany) to remove a stop codon and add a hexahistidine (His_6_) tag for affinity purification (pET-fp151).

### Co-expression of fp-151, tyrosinase, and ORF 438

The constructed recombinant plasmids, pET-fp151 and pACYC-Tyr-438, were introduced into *E. coli* BL21 (DE3) cells by electroporation for the co-expression of recombinant fp-151, tyrosinase, and ORF 438. A single colony from a freshly streaked plate of the cells was grown in 50 mL of LB medium with 50 μg/mL ampicillin and 10 μg/mL chloroamphenicol at 37°C overnight. 5 mL of the culture was transferred into 500 mL of LB medium with 50 μg/mL ampicillin and 10 μg/mL chloroamphenicol in a 3 L baffled flask, and cultivated at 37°C and 250 rpm until an OD_600_ of 0.8~1.0. Isopropyl-ß-D-thiogalactopyranoside (IPTG; Sigma) (final concentration, 1 mM) was added for induction of proteins, and cells were further incubated at 37°C and 250 rpm for 4 h. As a control, *E. coli* harboring sole pET-fp151 was also cultured in the same condition. Cells were harvested by centrifugation at 10,000 *g* for 10 min at 4°C, and the pellets were stored at −80°C for further purification.

The cells were disrupted on ice for 20 min by sonic dismembrator (Fisher Scientific, Waltham, MA, USA) using a 3 sec pulse on and 7 sec cooling period between each burst. The lysate was centrifuged at 10,000 *g* for 10 min at 4°C. The supernatant was collected for soluble fraction analysis. The residual pellet was washed twice with distilled water (DW) and resuspended in the same volume of lysis buffer before the sonication, which was used as insoluble fraction.

### Purification of *co*fp-151

To purify the co-expressed fp-151 with tyrosinase, the cell pellet was resuspended in 5 mL of lysis buffer (50 mM sodium phosphate buffer, 300 mM NaCl, 10 mM imidazole, and 8 M urea; pH 7.0) per gram of wet weight. The cells were disrupted on ice for 20 min by sonic dismembrator (Fisher Scientific) using a 3 sec pulse on and 7 sec cooling period between each burst. The lysate was centrifuged at 10,000 *g* for 10 min at 4°C. The supernatant was collected and applied to a Ni-NTA resin (Qiagen, Hilden, Germany) for affinity purification. After incubation of the lysate-Ni-NTA mixture for 1 h, the mixture was loaded into a column. The resin was washed three times with 5 column volumes of wash buffer (50 mM sodium phosphate buffer, 300 mM NaCl, 30 mM imidazole, and 8 M urea; pH 6.0). Recombinant fp-151 protein was eluted using 0.5 M hydrochloric acid. The eluted protein solution was diluted 5 times using DW and dialyzed twice in DW using a dialysis membrane of 12,000~14,000 Da molecular weight cut off (Spectrum Laboratories, Rancho Dominguez, CA, USA). Finally, the purified *co*fp-151 protein was freeze-dried and stored at −80°C. The expression level and purity were monitored by sodium dodecyl sulfate-polyacrylamide gel electrophoresis (SDS-PAGE). Gel-Pro Analyzer^TM^ (Media Cybermetics Inc., Bethesda, MD, USA) was used to analyze the protein level. The protein concentration was assessed using the Bradford protein assay method (Bio-Rad, Hercules, CA, USA).

### NBT staining and LC-MS/MS analysis

The modifications of tyrosine residues were detected by redox-cycling staining with NBT and glycinate reagent [24]. For the control experiments, *m*fp-151 was prepared using mushroom tyrosinase (Sigma) based on our previous protocol [35]. Two microliters of 3 mg/mL fp-151, *m*fp-151, and *co*fp-151 were dropped onto a nitrocellulose transfer membrane (Protran BA83; Whatman®, GE healthcare, Little Chalfont, UK). The membrane was immersed in a NBT/glycinate solution (0.6 mg/mL NBT and 2 M potassium glycinate buffer; pH 10) and incubated at room temperature until a blue-purple color developed. The stained membrane was washed using 0.1 M sodium borate solution and DW.

For LC-MS/MS analysis, the purified *co*fp-151 protein (20 μg) was dissolved in a digestion solution (6 M urea and 40 mM ammonium bicarbonate dissolved in high-performance LC-grade water). Protein reduction was performed with 5 mM tris(2-carboxyethyl) phosphine hydrochloride for 1 h, followed by an alkylation step with 25 mM iodoacetamide in the dark for 30 min at room temperature. The sample was digested with 5 ng/mL sequencing-grade modified trypsin (Promega) for 16 h at 37°C. All digested samples were collected and desalted with C-18 spin columns (Thermo, Rockford, IL, USA). Tryptic peptides were repeatedly analyzed (5 times) by LC-MS/MS on an LTQ XL mass spectrometer (Thermo Finnigan, San Jose, CA, USA). LC-MS/MS data were analyzed by a computational proteomics analysis system using the X! tandem search engine [39] with the database from Uniprot [40].

### Measurement of bulk-scale adhesive strength

Shear strength was analyzed using aluminum adherends (10 mm in width × 100 mm in length) with a hole placed near the ends [12]. The adherends were etched with 5% (w/v) NaOH solution for 5 min at room temperature, washed with DW, and immersed in a HNO_3_ solution (30% (v/v)) for 1 min to eliminate the smut layer formed by etching. Finally, the adherends were prepared by washing with DW and drying in air at room temperature. To measure the adhesive strength of *co*fp-151, 10 μL of 5% acetic acid solution was placed at the end of each adherend. When 5 mg of the lyophilized *co*fp-151 powder was added to one solution drop, the powder was quickly dissolved in the solution which produced a highly viscous emulsion. The viscous solution was mixed and spread well on the adhesion area (10 mm × 10 mm) of an adherend with a spatula. Then, another adherend was placed on the *co*fp-151 solution-coated surface with the 10 mm × 10 mm overlap area. The samples were incubated at 37°C for 3 h in air before measurements. The shear strength was directly obtained with a universal material testing machine (Instron 3344; Instron, Norwood, MA, USA) with a 2000 N load cell. These adhesion studies were an adaptation of the ASTM D1002 standard method (ASTM International D1002-05, 2005). Each adhesion measurement was repeated at least 5 times and averaged for a given sample.

BSA (Sigma), lysozyme (Sigma), non-modified fp-151, and *m*fp-151 were used at 500 mg/mL for the control experiments. *m*fp-151 was prepared by *in vitro* modification of fp-151. fp-151 protein (2 mg/mL) was incubated overnight at 37°C in phosphate-buffered saline (PBS; 2.68 mM KCl, 13.7 mM NaCl, 1.47 mM KH_2_PO_4_, and 0.875 mM Na_2_HPO_4_) with 25 mM ascorbic acid as a reducing agent and with 50 μg/mL mushroom tyrosinase (Sigma). Then, the resultant *m*fp-151 was freezed-dried after the dialysis twice against DW.

## Competing interests

The authors declare that they have no competing interests.

## Author’s contributions

YSC and HJC designed research. YSC, YJY and BY performed the experiments and analyzed the results. YSC and HJC wrote the paper. All authors have read and approved final version of the manuscript.
